# Supplementary material for: RIDGE: Reproducibility, Integrity, Dependability, Generalizability, and Efficiency Assessment of Medical Image Segmentation Models
Source: J Imaging Inform Med. 2024 Nov 18;38(4):2524–36. doi: 10.1007/s10278-024-01282-9 (PMC12343378; doi:10.1007/s10278-024-01282-9)
Supplement: Supplementary file 1 — Supplementary file1 (DOCX 18 KB) [file 10278_2024_1282_MOESM1_ESM.docx]

Table S1. The RIDGE Checklist builds upon the best practices and guidelines already highlighted by other checklists, such as CLAIM, STARD, and TRIPOD.

| RIDGE | CLAIM | STARD | TRIPOD |  | RIDGE | CLAIM | STARD | TRIPOD |
| --- | --- | --- | --- | --- | --- | --- | --- | --- |
| I-1 | ✓ | ✓ | ✓ |  | M-22 | ✓ |  |  |
| I-2 | ✓ | ✓ | ✓ |  | M-23 | ✓ |  |  |
| M-1 | ✓ | ✓ | ✓ |  | M-24 |  |  |  |
| M-2 | ✓ | ✓ | ✓ |  | M-25 |  |  |  |
| M-3 | ✓ | ✓ | ✓ |  | M-26 |  |  |  |
| M-4 | ✓ | ✓ | ✓ |  | M-27 | ✓ | ✓ |  |
| M-5 | ✓ | ✓ | ✓ |  | M-28 |  |  |  |
| M-6 | ✓ | ✓ |  |  | M-29 | ✓ |  |  |
| M-7 | ✓ | ✓ |  |  | M-30 |  |  |  |
| M-8 | ✓ |  |  |  | R-1 | ✓ |  |  |
| M-9 | ✓ |  |  |  | R-2 |  |  |  |
| M-10 | ✓ |  | ✓ |  | R-3 |  |  |  |
| M-11 | ✓ | ✓ | ✓ |  | R-4 |  |  |  |
| M-12 | ✓ |  |  |  | R-5 |  |  |  |
| M-13 | ✓ |  |  |  | R-6 |  |  |  |
| M-14 | ✓ |  |  |  | D-1 | ✓ | ✓ | ✓ |
| M-15 | ✓ |  |  |  | D-2 | ✓ | ✓ |  |
| M-16 | ✓ |  |  |  | D-3 |  |  |  |
| M-17 | ✓ |  |  |  | C-1 |  |  |  |
| M-18 | ✓ |  |  |  | C-2 |  |  |  |
| M-19 | ✓ | ✓ | ✓ |  | C-3 |  |  |  |
| M-20 | ✓ | ✓ |  |  | C-4 |  |  |  |
| M-21 | ✓ |  |  |  | S-1 |  |  |  |
